# Clinical audit of cardio-metabolic monitoring in people with intellectual disability (PWID) taking antipsychotic medication

**DOI:** 10.1192/bjo.2021.268

**Published:** 2021-06-18

**Authors:** Sandar Kyaw, Fadzlien Bintizahari, Peter Speight

**Affiliations:** Lincolnshire Partnership Foundation Trust

## Abstract

**Aims:**

To ensure close monitoring of physical health parameters when antipsychotics are prescribed and to liaise with primary care to ensure appropriate interventions are implemented.

**Background:**

Antipsychotics are the most frequently prescribed psychotropic medication for PwID. Treatment with antipsychotic agent is associated with cardio-metabolic risks such as obesity, diabetes, and dyslipidemia. A strong association is well documented between antipsychotic use and the risk of stroke in schizophrenia although the magnitude of this association has yet to be studied in PwID.

PwID have an increased risk of premature death. Cardio-metabolic monitoring and appropriate intervention to this vulnerable cohort will improve the preventable cardio-metabolic multi-morbidity. The NICE guideline (CG11) recommends antipsychotic medication should only be initially prescribed and monitored by the secondary care professionals for at least 12 months. They also should work together with primary care to ensure appropriate interventions are arranged where necessary.

**Method:**

A retrospective audit was performed for 40 service users, taking antipsychotic medication. Quota sampling was used to identify 10 cases each from the caseload of 4 consultant psychiatrists, within the Intellectual Disability community setting, between September 2019 and October 2019.

An audit tool was designed, in accordance with cardio-metabolic measures (smoking status, height, weight, Blood Pressure, HbA1c, Lipid profile), based on physical health CQUIN targets and the Lester adaptation tool. Collection of data was performed from electronic case records and electronic blood results service. The work was performed with the approval of local clinical audit team and analysed by using Microsoft Excel.

**Result:**

Baseline cardio-metabolic assessment was observed in over a half of the sample population (50–65%) whilst only less than 15% was noted at 3–6 months. Documentation Evaluation of physical health assessments for new admissions to the Oleaster during the first wave of COVID-19 on body weight and blood pressure was seen only in 15% and 2.5% of population respectively at 3–6 months. Collaboration with GP for annual health check was observed in 78–100% of population.

Intriguingly, our finding indicates a significant improvement in all required compliance when nursing team is involved.

**Conclusion:**

Improving physical healthcare is essential to reduce the cardio metabolic outcome in PwID taking antipsychotic medication. Better involvement of community nurses as well as availability of Sphygmomanometers at every outpatient clinic will determine the successful implementation of cardio metabolic monitoring and effective collaboration with primary care clinicians.

Once the action plan is disseminated to the teams, the impact of change will be reassessed by a re-audit in one year's time.

